# Special Issue “An Update on Lactobacillus”: Editorial

**DOI:** 10.3390/microorganisms11061400

**Published:** 2023-05-26

**Authors:** Piotr Heczko, Łucja Kozień, Magdalena Strus

**Affiliations:** Chair of Microbiology, Department of Bacteriology, Microbial Ecology and Parasitology, Jagiellonian University Medical College, 31-121 Cracow, Poland

As indicated in the introduction to this Special Issue, as of 2020, the original genus *Lactobacillus* comprised over 260 recognized species, a figure which is probably much higher now. These species are extremely diverse at the phenotypic, ecological, and genotypic levels. Therefore, a new taxonomy within the *Lactobacillaceae* family has been proposed, and now the former genus *Lactobacillus* has been re-classified into 25 genera, with the addition of 23 novel genera. Fortunately for all researchers working on different questions related to medicine, nutrition, etc., the generic terms *Lactobacillus* and ‘lactobacilli’ will remain useful to designate all organisms that were classified as *Lactobacillaceae* until 2020 [1].

Most of the evidence supporting the idea of the leading role of the *Lactobacillus* genus in the housekeeping of human and animal health derives from articles on probiotics aimed at elucidating the mechanisms of their functional activities. Therefore, the terms *Lactobacillus* and probiotic bacteria are often regarded as synonymous, which should be avoided in research articles. It is, of course, not possible to expand the data obtained in studies of probiotic strains regarding all of the *Lactobacillus* strains that are contact with human body surfaces and/or anchored in various ecological niches. It is, however, possible to speculate that many properties which have been attributed to the individual probiotic *Lactobacillus* may be a common characteristic of the whole species. Historically, probiotic lactobacilli were isolated at random from different niches of the healthy human microbiome and then characterized; therefore, it is highly probable that there are many *Lactobacillus* strains sharing the same properties as well-known probiotic strains that are active in the human microbiome but remain undetected/uncharacterized [2].

Accumulating data demonstrate that the gut microbiome contributes to early-life imprinting, particularly through its effects on the developing immune system [3]. Although the underlying molecular mechanisms of this neonatal priming period in humans have not been defined, thanks to new animal experiments, there are new data showing that the mechanisms of acquiring the gut microbiota in infancy depend on interactions between bacterial and host factors. This suggests that the timing of bacterial arrival in the gut is very important in shaping the gut microbiome. This is the case for the *Lactobacillus* bacteria: they form the dominant part of the vaginal microbiota in the late period of pregnancy, but they are also present in high numbers in human milk [4]. Thus, lactobacilli numerically overshadow all other genera, including *Bifidobacterium*, in colonizing neonatal mucosal and skin surfaces at birth during passage though the vagina and its *Lactobacillus*-rich microbiota, and then after labour during feeding, with the mother’s breast milk containing high numbers of lactobacilli [5]. Thus, proper timing and proper bacteria are the crucial factors that may determine the successful artificial colonization of neonates at risk [6]. In spite of a large number of randomized placebo-controlled clinical trials and observational cohort studies including more than 50,000 preterm infants from 29 countries that have demonstrated a decrease in the risk of necrotizing enterocolitis, death, and sepsis, routine prophylactic probiotic administration to preterm infants remains uncommon in much of the world [7]. An article published in this Special Issue presents new data regarding the successful colonization of extremely preterm neonates after supplementation with a new strain of *Limosilactobacillus reuteri* [8], although this species was previously considered as less colonization-efficient in comparison with others [9].

On the contrary to the above, *Lacticaseibacillus paracasei* strain Shirota has been the object of over 500 scientific studies and is considered as one of the most researched probiotic strains, originally selected in 1930 by Doctor Minoru Shirota, and fully characterized and commercialized about twenty years later. The discovery of the gut–brain axis prompted researchers to study its mechanisms and the effectiveness of *Lactobacillus* probiotics in ameliorating depressive symptoms. The gut–brain axis refers to bidirectional communication between the brain and the gut, and is related to alterations in the gut microbiota composition [10]. Furthermore, *L. paracasei* Shirota strain was also investigated in clinical studies to check its anti-depressive activity [11]. The intervention-associated reduction in depressive symptoms was associated with the gut microbiota, and was more pronounced when *Bifidobacterium* and *Atopobium* clusters of the *Actinobacteria* phylum were maintained at higher counts. 

It is well documented that the human vaginal microbiota is composed of several dozens of bacterial species, with a distinct predominance of several *Lactobacillus* strains efficiently controlling the remaining members of the microbiota by direct means, i.e., the production of lactic and other acids able to kill other bacteria [12,13]. 

However, it is not yet known if and how the dominant lactobacilli control atypical bacteria that are not members of the microbiota and invade the vagina as result of sexual contacts, as *Chlamydia trachomatis* does [14]. The literature on this subject is rather scanty and different mechanisms are proposed: the induction of anti-inflammatory cytokines [15] or the expression of α5β1 integrin in cervical cells [16], and more recently, the production of biosurfactant by *Lactobacillus crispatus*, as published and presented in this Special Issue [17]. 

There is also a rapidly accumulating bulk of the literature that is focused on lactobacilli in human and animal foods; in fact, this large research area is also strictly related to health, and contains important and valuable information for industry both in the technological and economic sense. Thus, it is worth indicating here that *Lactobacillaceae* are the most often domesticated bacteria for nourishment. During the domestication process, microbes gained the capacity to efficiently consume particular nutrients, cope with a multitude of industry-specific stress factors, and produce desirable compounds, often at the cost of a reduction in fitness in their original, natural environments [18]. Historically, lactobacilli fermenting a practically unlimited varieties of plants, dairy products, fish, and meat were recognized as useful bacteria just after the discovery of the microbial world in the last century. In this way, examples such as the domesticated yoghurt producer *Lactobacillus delbrueckii* ssp. *bulgaricus* or the meat- and fish-fermenting *Latilactobacillus sakei* were discovered and characterized [19,20]. Discoveries of new domesticated *Lactobacillus* species are announced continuously. Moreover, the availability of whole-genome sequencing data, combined with an expansive experimental toolbox, allows researchers to generate novel, superior variants in the laboratory [18]. A very good example of this new approach to the domestication of the industrially important *Lactiplantibacillus plantarum* is presented in this Special Issue [21]. This example shows that although *L. plantarum* bacteria does not readily utilize plant fructo-oligosaccharides, they may create them efficiently in the presence of cranberry polyphenols. This may provide next-generation synergistic symbiotic approaches that incorporate adjunct substrates such as cranberry polyphenols. Cranberries are often used in polyphenol-enriched food products, which have been reported to be effective in addressing obesity, inflammation, and cardiovascular disease, and more specifically have been included in dietary supplements used to prevent urinary tract infections, since they inhibit the adhesion of uropathogenic *Escherichia coli* to the urinary epithelium [22]. 

## Data Availability

Not applicable.
